# Smooth pursuit adaptation (SPA) exhibits features useful to compensate changes in the properties of the smooth pursuit eye movement system due to usage

**DOI:** 10.3389/fnsys.2013.00067

**Published:** 2013-10-17

**Authors:** Suryadeep Dash, Peter Thier

**Affiliations:** ^1^Department of Cognitive Neurology, Hertie Institute for Clinical Brain Research, Center for Neurology, University of Tübingen, Tübingen, Germany; ^2^Robarts Research Institute, Western University, London, ON, Canada

**Keywords:** smooth pursuit, monkey, adaptation, ocular, fatigue, vermis

## Abstract

Smooth-pursuit adaptation (SPA) refers to the fact that pursuit gain in the early, still open-loop response phase of the pursuit eye movement can be adjusted based on experience. For instance, if the target moves initially at a constant velocity for ~100–200 ms and then steps to a higher velocity, subjects learn to up-regulate the pursuit gain associated with the initial target velocity (gain-increase SPA) in order to reduce the retinal error resulting from the velocity step. Correspondingly, a step to a lower target velocity leads to a decrease in gain (gain-decrease SPA). In this study we demonstrate that the increase in peak eye velocity during gain-increase SPA is a consequence of expanding the duration of the eye acceleration profile while the decrease in peak velocity during gain-decrease SPA results from reduced peak eye acceleration but unaltered duration. Furthermore, we show that carrying out stereotypical smooth pursuit eye movements elicited by constant velocity target ramps for several hundred trials (=test of pursuit resilience) leads to a clear drop in initial peak acceleration, a reflection of oculomotor and/or cognitive fatigue. However, this drop in acceleration gets compensated by an increase in the duration of the acceleration profile, thereby keeping initial pursuit gain constant. The compensatory expansion of the acceleration profile in the pursuit resilience experiment is reminiscent of the one leading to gain-increase SPA, suggesting that both processes tap one and the same neuronal mechanism warranting a precise acceleration-duration trade-off. Finally, we show that the ability to adjust acceleration duration during pursuit resilience depends on the integrity of the oculomotor vermis (OMV) as indicated by the complete loss of the duration adjustment following a surgical lesion of the OMV in one rhesus monkey we could study.

## Introduction

Smooth pursuit eye movements (SPEM) are used to stabilize the image of a moving object of interest on the fovea, thus allowing the observer to deploy the advantages of foveal vision for the scrutiny of the object in motion. SPEM are driven by the motion of the retinal target image which is translated into an appropriate eye movement response, reducing target image slip in a closed-loop manner (Rashbass, 1961; Robinson et al., 1986). However, due to the latencies of visual information processing, the eye movement response becomes available only 100–150 ms after target motion onset. Correspondingly, the first 100–150 ms of the SPEM are driven by uncompensated retinal target image motion. In other words, they reflect an open-loop response whose size depends solely on the visual target motion signal and a gain parameter that specifies the mapping of the visual information onto the motor response. Smooth pursuit adaptation (SPA) refers to the fact that the gain of SPEM initiation can be adjusted by suitable experimental manipulations. Under laboratory conditions, adaptation can be easily demonstrated by deploying changes in target velocity around the time the open-loop response phase ends. Initially, the target moves at a constant velocity for ~100–200 ms whereupon it steps to a different velocity. In reaction to these velocity steps initial SPEM velocity changes such as to draw eye velocity evoked by the initial target velocity nearer to the target velocity after the velocity step. This phenomenon is called SPA (Fukushima et al., 1996; Kahlon and Lisberger, 1996; Dash et al., 2010). If the target moves initially at a constant velocity for roughly 100–200 ms and then steps to a higher velocity, subjects learn to up-regulate the pursuit gain evoked by the initial target velocity (gain-increase SPA). Correspondingly, a step to a lower target velocity leads to a decrease in gain (gain-decrease SPA). Yet, the stereotypic steps in target velocity able to evoke SPA hardly occur outside the laboratory. This leads to the question what the ecological role of SPA might be? The standard answer to this question is that SPA reflects a mechanism needed to cope with changes in the visuo-motor mapping required by development or disease. Clearly SPEM can adjust to such long-term changes. For instance, if patients with one paretic eye are asked to view with that eye while the healthy one is covered, after a few days of habituation, the normal eye may exhibit an increased SPEM gain (Optican et al., 1985). However, one may legitimately doubt that such adjustments would require a mechanism, which is as fast as SPA, which typically unfolds over a few dozen trials of smooth-pursuit only. Here we provide behavioral evidence supporting the notion that SPA reflects the working of a mechanism whose functional role is the compensation of *fatigue*, the latter term serving as an umbrella for the manifold factors changing eye movement performance due to usage on a comparatively short time scale. This conclusion is based on a quantitative comparison of eye movement kinematics of monkeys during SPA and during repetitive normal, unadapted SPEM.

Lesions of vermal lobuli VI and VIIa (=oculomotor vermis or OMV) are known to impair SPA (Takagi et al., 2000). We hypothesized that this deficit might be a consequence of a loss of the acceleration-duration trade-off described before. In order to test this hypothesis, we performed a surgical lesion of the OMV in one rhesus monkey. In full accordance with our expectation we observed that the lesioned monkey was unable to sustain an appropriate level of pursuit velocity in the resilience task due to his inability to upregulate acceleration duration in order to compensate the unavoidable decrease in peak acceleration.

## Methods

### Animal procedures

Two male rhesus (*Macaca mulata*) monkeys (E and S) were used in this study. They were prepared for eye position recording using the magnetic search coil technique (Judge et al., 1980). They were implanted with a titanium head post for the painless immobilization of the head. Details of surgical procedures and post-surgical care are explained elsewhere (Ignashchenkova et al., 2009). All procedures complied with the NIH Guide for Care and Use of Laboratory Animals and were approved by the local animal care committee.

The monkeys were trained to generate the behavior of interest by rewarding them with units of fluid (juice or water, the latter if preferred by the monkey), needed to satisfy their daily fluid requirements. Careful monitoring of fluid intake and body weight and supplementation of fluid outside the experiment if needed ensured that the animals were sufficiently hydrated at any time.

### Behavioral procedures

The monkeys were trained to keep their line of sight within an eye position window of 2–3° diameter centered on the fixation target (3 min of arc diameter) presented on a computer monitor (Mitsubishi, 50 cm screen diagonal, frame rate 72 Hz, 1280 × 1024 pixels) placed 43 cm in front of the monkeys in an otherwise completely dark room. To elicit pursuit eye movements, we used a step-ramp sequence, consisting of an initial target step away from the central fixation point in a direction opposite to the direction of the subsequent target ramp. The step amplitude depended on the ramp velocity and the pursuit latency of the individual monkey and was chosen such as to have the target back at straight ahead at pursuit onset, thereby minimizing the need for catch-up saccades (Rashbass, 1961). Individual trials started after a preceding fixation period, whose duration was varied between 500 and 800 ms. SPA was induced by introducing a change in target velocity, when the target reached the straight ahead position following the step back. On different experimental sessions, target velocity was either decreased (from 20 to 5°/s; “gain-decrease SPA”) or increased (from 10 to 40°/s; “gain-increase SPA”). Both forms of SPA usually needed 75–200 trials. Experimental sessions in which we tried to reveal changes due to continuous generation of unadapted SPEM (“SPEM resilience” experiments) comprised of target ramps presented at constant velocity of 18.5°/s for 150–500 trials in quick succession. The choice of velocity configurations used for both gain-decrease SPA and gain-increase SPA was motivated by the good performance of the monkeys for these configurations. The choice of 18.5°/s for the SPEM resilience experiment was arbitrary.

Trial history as well as the eye position records sampled at 1 kHz were stored for offline analysis. The analysis was carried out using self-written MATLAB programs (MATLAB, The MathsWorks Inc., MA). The recorded horizontal and vertical eye position traces were first smoothed with a Savitzky-Golay filter (window = 10 points, polynomial degree = 4), which replaces the data points in the specified window by a polynomial regression fit of the chosen degree. Instantaneous eye velocity was derived from the filtered eye position records by differentiating the eye position and eye acceleration was derived by differentiating eye velocity records. Pursuit onset was determined by identifying a significant change in the eye velocity record starting from target onset toward later points in time where eye velocity first exceeded 2 times the standard deviation of eye velocity during fixation (=baseline eye velocity). Trials with saccades during the initial 175 ms of eye movement as well as trials with a pursuit latency of more than 200 ms were not considered for further analysis. For none of the sessions included in this study, the rejected trials exceeded more than 5% of the total number of trials. For every trial we calculated the maximum eye velocity in the first 175 ms and for each experiment we compared the average of the maximum eye velocity for the first quarter with the one for the last quarter of the trials. We determined the maximum eye velocity in the 175 ms instead of the first 100 ms as in previous work as in our material peak velocity was rarely attained within the first 100 ms but in many cases required up to 150–200 ms to be reached. Moreover, as will be clear from the results, the kinematic changes associated with adaptation extended beyond 100 ms following pursuit onset. The SPA was considered significant (*t*-test, *p*-value < 0.05) if the average peak eye velocity increased or decreased significantly between the first and the last quarter of the trials during gain-increase and gain-decrease SPA, respectively, and only those sessions were considered for further analysis. However, when we repeated the same analysis considering the maximum velocity at 100 ms, all the sessions included in either form of SPA showed significant adaptation effects. Well-trained monkey subjects rather than human volunteers were used in these experiments because of the lower variability in performance as well as the possibility to study the consequences of a targeted cerebellar lesion.

### Cerebellar lesion

The posterior cerebellar vermis was lesioned in monkey S in two stages, once sufficient pre-lesion data had been collected. The surgical procedure and post-surgical care of the monkey are described in detail elsewhere (Barash et al., 1999; Ignashchenkova et al., 2009). Immediately after a first lesion (L1), whose extent and boundaries are described below, the monkey showed saccadic hypometria, one of the hallmarks of acute lesions of the OMV (Takagi et al., 1998; Barash et al., 1999). However, measurements of spontaneous eye movements performed while the monkey was being cleaned showed that recovery from hypometria was unusually fast with complete normalization of saccade metrics already 4 days after the lesion. Moreover, spontaneous smooth pursuit eye movement did not seem to be impaired at any point. High-resolution anatomical MRI showed that the pettiness and transiency of the oculomotor disturbances were a consequence of the fact that the lesion had largely missed the OMV proper. As shown in Figure 4A, the lesion encompassed lobulus VIII and caudal VII but spared the more rostral parts of lobulus VII and neighboring lobulus VI.

We collected 4 sessions of SPEM resilience data 20 days after L1 and then performed a second ablation (L2) aiming at destroying lobuli VI and rostral VII 36 days after L1. A subsequent high resolution MRI confirmed that L2 indeed included lobuli VI and rostral VII as well as lobulus V (Figure 4B). In accordance with the now complete loss of the OMV, the monkey exhibited the typical signs of acute OMV lesion such as saccadic hypometria. A reconstruction of the cumulative vermal lesions based on post-mortem histology is shown in a recent report on the role of the OMV in various cognitive tasks, a study in which monkey S served as one of the subjects (Ignashchenkova et al., 2009). Monkey E is still being used in unrelated experiments.

## Results

**Figure 1:**
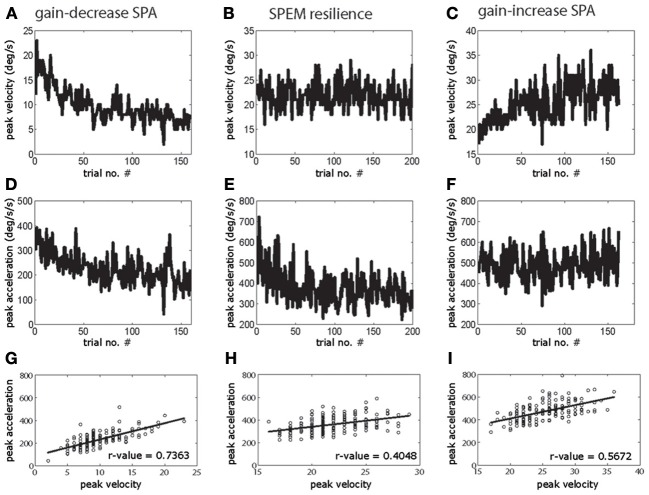
**Exemplary sessions of gain-decrease SPA (left column), SPEM resilience (middle column) and gain-increase SPA (right column)**. The upper row shows the changes in peak eye velocity **(A–C)** during the course of sessions, the middle row depicts the accompanying changes in peak acceleration **(D–F)**. The lower row **(G–I)** shows the correlation between peak velocity and peak acceleration on a trial to trial basis.

Figure 1 shows examples of individual sessions of gain-increase SPA, SPEM resilience, and gain-decrease SPA, demonstrating the characteristic progression of peak eye velocity changes and the associated changes in peak acceleration. Clearly, peak velocity drops during gain-decrease SPA (*t*-test, *p* < 0.05, comparison of first and last quarter of the trials; Figure 1A) which is a consequence of a drop in peak acceleration (*t*-test, *p* < 0.05, comparison of first and last quarter of the trials; Figure 1D). However, corresponding yoked changes in peak velocity and acceleration were not observed during SPEM resilience and gain-increase SPA. During SPEM resilience peak velocity was maintained (*t*-test, *p* > 0.05, comparison of first and last quarter of the trials; Figure 1B), despite the fact that there was a drop in peak acceleration (*t*-test, *p* < 0.05; Figure 1E). Finally, during gain-increase SPA peak velocity increased (*t*-test, *p* < 0.05; Figure 1C), whereas peak acceleration did not show a corresponding increase (*t*-test, *p* > 0.05; Figure 1F). These exemplary observations suggest that gain-decrease and gain-increase SPA are not simply based on mirror symmetric adaptive mechanisms. On the other hand, the comparable changes in peak acceleration exhibited by gain-decrease SPA and SPEM resilience might actually indicate a functional commonality. The above results for a single session were consistent across all the sessions in both animals. Peak velocity and peak acceleration decreased in all the gain-decrease SPA sessions (*t*-test, *p* < 0.05, comparison between first and last quarter of the trials). During all the SPEM resilience sessions, the peak velocity did not change during the course of the session (*t*-test > 0.05), while peak acceleration decreased invariably in every single session (*t*-test, *p* < 0.05). Gain-increase SPA showed changes in peak acceleration which were inconsistent across sessions. While all the sessions exhibited a clear increase in peak velocity (*t*-test, *p* < 0.05), some sessions showed an increase in peak acceleration (*n* = 7 sessions, *t*-test, *p* < 0.05), whereas others remained without change in peak acceleration (*n* = 10 sessions, *t*-test, *p* > 0.05). Some even showed a decrease in peak acceleration (*n* = 4 sessions, *t*-test, *p* < 0.05). The above analysis could suggest that during gain-decrease SPA, a drop in peak acceleration determines the decrease in pursuit peak velocity. If this were true then peak acceleration and peak velocity should co-modulate in a trial-by-trial fashion. Figure 1G indeed shows that the trial-by-trial relationship between peak velocity and peak acceleration was characterized by significantly larger coefficients of correlation during gain-decrease SPA when compared to either SPEM resilience or gain-increase SPA (Figures 1H,I). This robust correlation between peak velocity and peak acceleration during gain-decrease SPA was true for all the sessions from both monkeys. The mean correlation coefficient between peak velocity and peak acceleration for all the sessions with gain-decrease SPA (mean *r*-value = 0.692) was significantly higher than for either SPEM resilience (mean *r*-value = 0.47) or gain-increase SPA (mean *r*-value = 0.451) (*t*-test, *p* < 0.05, corrected for multiple comparison).

**Figure 2:**
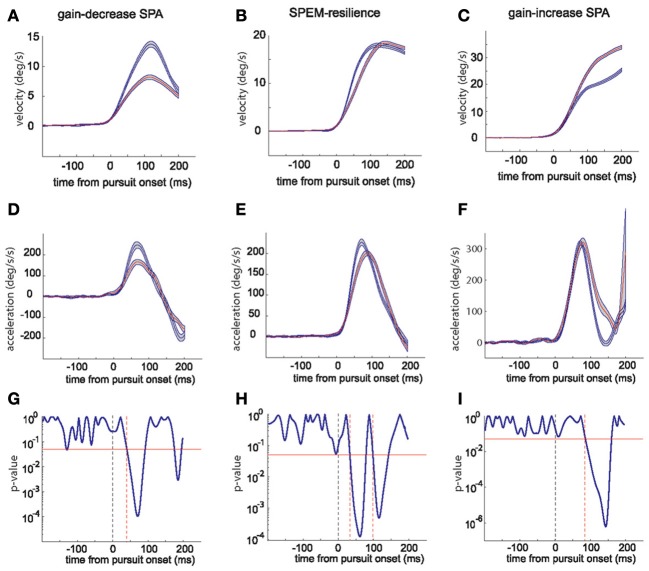
**The top row panels show the average eye velocity in the first quarter (blue) and last quarter (red) of trials during a typical gain-decrease SPA session (A), SPEM resilience session (B) and gain-increase SPA session (C)**. The middle row panels **(D–F)** depict the corresponding average eye acceleration traces. The gray shadow around the red and blue traces signifies the standard error. The lower row panels **(G–I)** show the periods when the acceleration profiles [blue and red; **(D–F)**] were significantly different from each other based on a running *t*-test. The red horizontal line indicates a *p*-value of 0.05. The dashed vertical (black) line shows pursuit onset and the red vertical lines give the time points when the significance level crossed the *p*-value of 0.05.

The changes in peak velocity and peak acceleration suggested by the exemplary sessions are clearly supported by a comparison of the average kinematic profiles based on mean eye velocity and acceleration records for the first and the last quarter of trials in a given session (see Figure 2 for examples). These averages, moreover, reveal important temporal information that allows one to understand the distinct combinations of peak eye velocity and acceleration varying for the three paradigms. During gain-decrease SPA the drop in peak velocity (Figure 2A; blue represents average of first quarter of trials and red represents the last quarter) was due to a decrease in the initial acceleration peak, whose duration did not change (Figure 2D). On the other hand, the increase in peak velocity during gain-increase SPA (Figure 2C) was a consequence of an expansion of the acceleration profile (Figure 2F), whereas peak acceleration did not change significantly. The SPEM resilience session showed no change in peak velocity (Figure 2B) but changes in the acceleration profile which combined features of both gain-decrease SPA and gain-increase SPA (Figure 2E). Similar to gain-decrease SPA the peak acceleration dropped during the course of the session and analogous to gain-increase SPA an expansion in the duration of the acceleration profile was observed.

To delineate the occurrences of differences in the acceleration profiles more precisely, we deployed a running *t*-test with a sliding time window of 5 ms, which compared the mean profiles for the first and the last quarters of trials. This statistical analysis revealed that the difference between early and late acceleration profiles appeared much earlier for gain-decrease SPA (around 40 ms after pursuit onset; Figure 2G) than for gain-increase SPA (around 90 ms after pursuit onset; Figure 2I). The differences in acceleration profiles characterizing SPEM resilience are a concatenation of the ones for gain decrease and increase SPA (Figure 2H): an early significant difference due to a drop in peak acceleration (around 40 ms after pursuit onset, similar to gain-decrease SPA) was followed by a late period of significant difference (100 ms), reflecting the compensatory expansion of the acceleration profile (similar to gain-increase SPA). All the gain-decrease SPA sessions (18 sessions from monkeys E and S) and all the SPEM resilience sessions (12 sessions from monkeys E and S) showed patterns similar to the one shown in Figures 2G,H, respectively. However, all the gain-increase SPA sessions showed the drop of the *p*-value around 75–100 ms as exemplified in Figure 2I, lasting till 150–180 ms after pursuit onset, signifying the expansion of the acceleration profile; with some sessions showing an additional early period of a significance difference (*p*-value going below 0.05), indicating an increase in peak acceleration (*n* = 7) or a drop in peak acceleration (*n* = 4).

**Figure 3:**
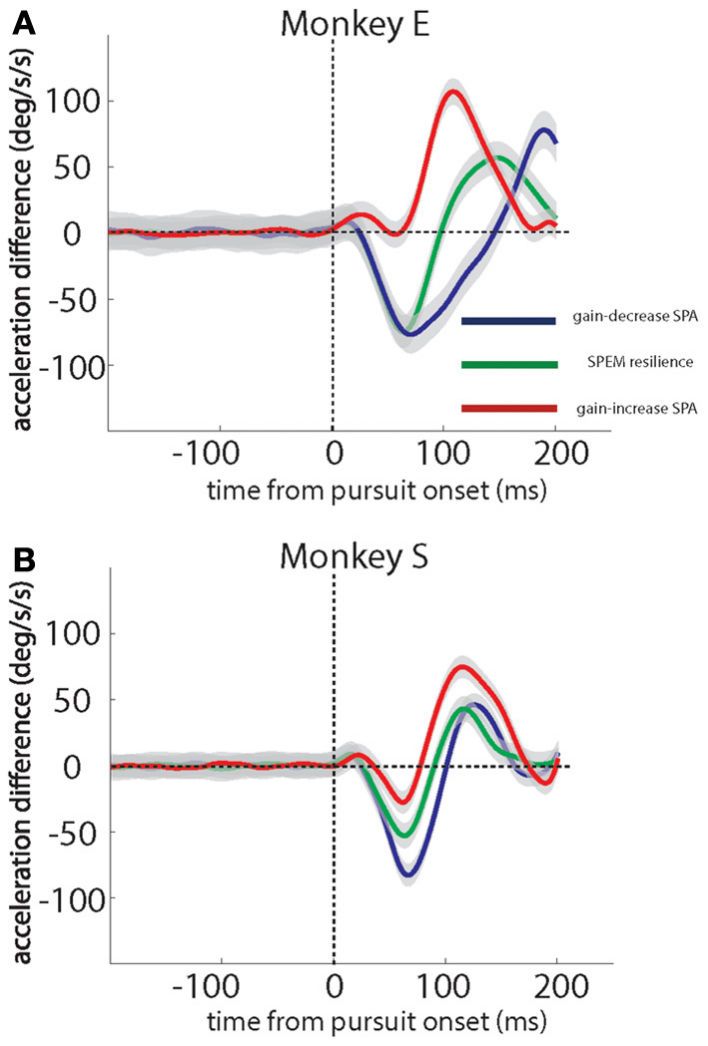
**Grand averages of eye acceleration profile differences based on a comparison of the first and the last quarter of trials in individual sessions for the 2 monkeys studied**. Gain-decrease SPA (blue), SPEM resilience (green) and gain-increase SPA (red). Data for monkey E in **(A)**, for monkey S in **(B)**. Monkey E and monkey S contributed 7 and 11 sessions of gain-decrease SPA, respectively, 7 and 5 sessions of SPEM resilience, respectively, and 11 and 10 sessions of gain-increase SPA, respectively. The gray shadows around the traces signify standard error.

To confirm the generality of the kinematic changes observed in the exemplary sessions, we calculated the differences between the mean acceleration profiles for the first quarter of trials and the last quarter of the trials for each session and averaged the resulting differences across sessions. Figures 3A,B show these grand averages for monkey E and monkey S, respectively. They are in full accordance with the patterns characterizing individual sessions described before. During gain-decrease SPA (blue traces), both monkeys exhibited an early negative acceleration difference (20–30 ms after pursuit onset) peaking around 75–80 ms after pursuit onset, reflecting the loss of acceleration in the course of the sessions. On the other hand, during gain-increase SPA (red traces), both monkeys E and S showed a later positive peak in the difference curve as a consequence of the gradual expansion of the acceleration profile (Figures 3A,B). This acceleration burst started around 60–80 ms after SPEM onset and continued until around 175 ms after SPEM onset. In monkey S it was preceded by an early small decline in acceleration between 35–75 ms after SPEM onset, followed by a sharp increase in the acceleration difference at around 75–175 ms after SPEM onset. Finally, SPEM resilience (green traces) exhibited the combination of the features of both gain-decrease SPA and gain-increase SPA. In both monkeys we observed an early negative peak in the acceleration difference which was later followed by a compensatory increase.

**Figure 4:**
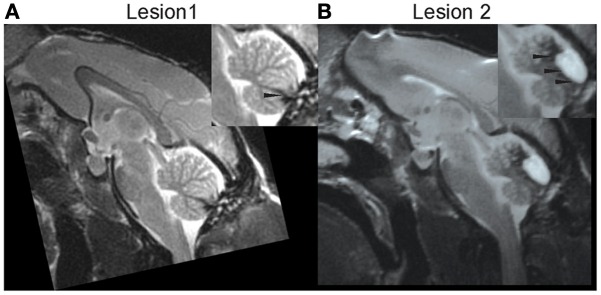
**High resolution midline sagittal MRI slice after lesion 1 (A) and after lesion 2 (B)**. Adopted from Ignashchenkova etal. (2009). Further technical details are provided there.

**Figure 5:**
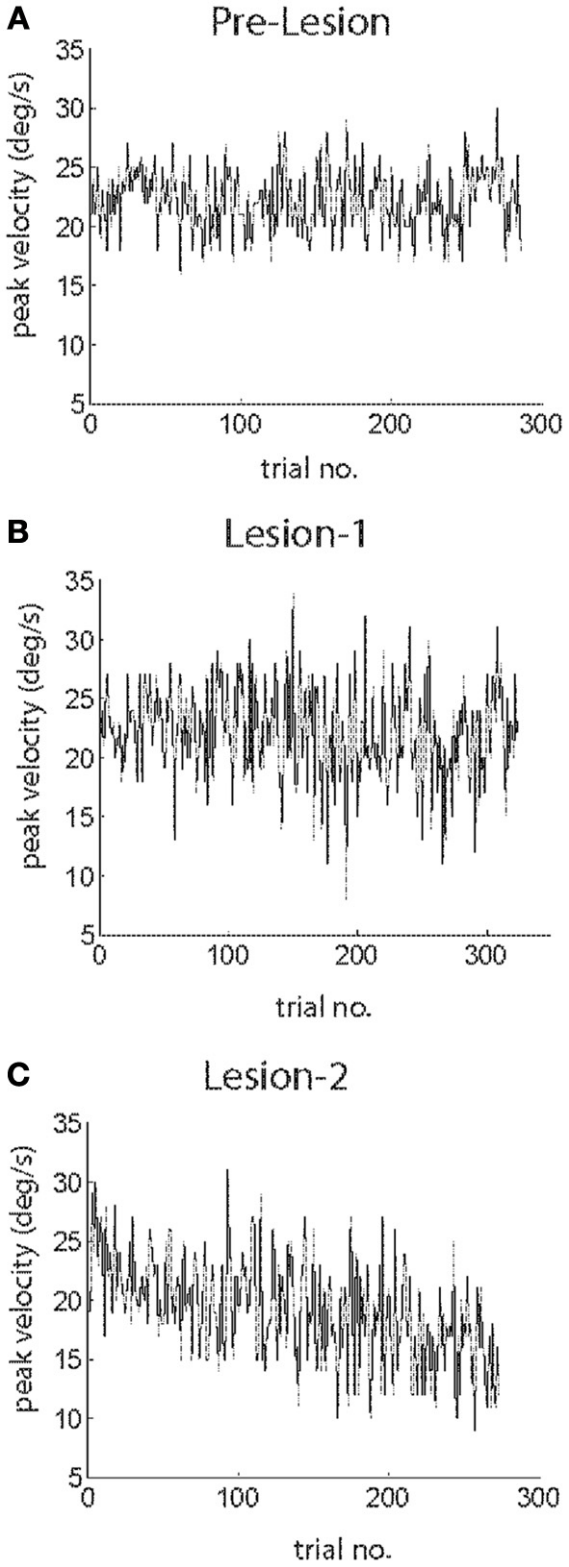
**Plots of peak eye velocity as function of trial number for exemplary sessions of SPEM resilience before the lesion (A), after L1 (B) and after L2 (C)**. Note that eye velocity declines with increasing trial number after L2.

Monkey S also participated in a subsequent lesion study on cerebellar contributions to visual perception summarized in Ignashchenkova et al., 2009. We were fortunate to collect a few post-lesion SPEM resilience sessions. Figures 5A–C show plots of peak velocity as function of trial numbers for representative SPEM resilience experiments before the lesion **(A)**, 20 days after L1 **(B)** and 45 days after L2 **(C)**. Before any lesion, the animal was able to keep the peak pursuit velocity constant throughout the course of the SPEM resilience experiment (Figure 5A). Comparison of peak velocity in the first and the last quarter of trials did not reveal any difference (*t*-test; *p* > 0.05 for all 5 SPEM resilience sessions). Figure 5B shows one of the two sessions recorded after L1, which likewise revealed no change in the peak velocity during the course of the SPEM resilience experiment (comparison of 1st and 4th quarter by *t*-test, *p* > 0.05). After L2, peak velocity was normal early in the experiment (comparison of the mean peak velocity for the 1st quarter of trials pre-lesion, after L1 and after L2 by One-Way ANOVA, *p* > 0.05). However, after L2 the peak velocity declined significantly in the course of the resilience session (Figure 5C; comparison of 1st and 4th quarter for all 6 SPEM resilience sessions by *t*-test, *p* < 0.05). To further explore the nature of this instability of peak velocity observed after L2 we next took a closer looked at the temporal structure of pursuit initiation.

**Figure 6:**
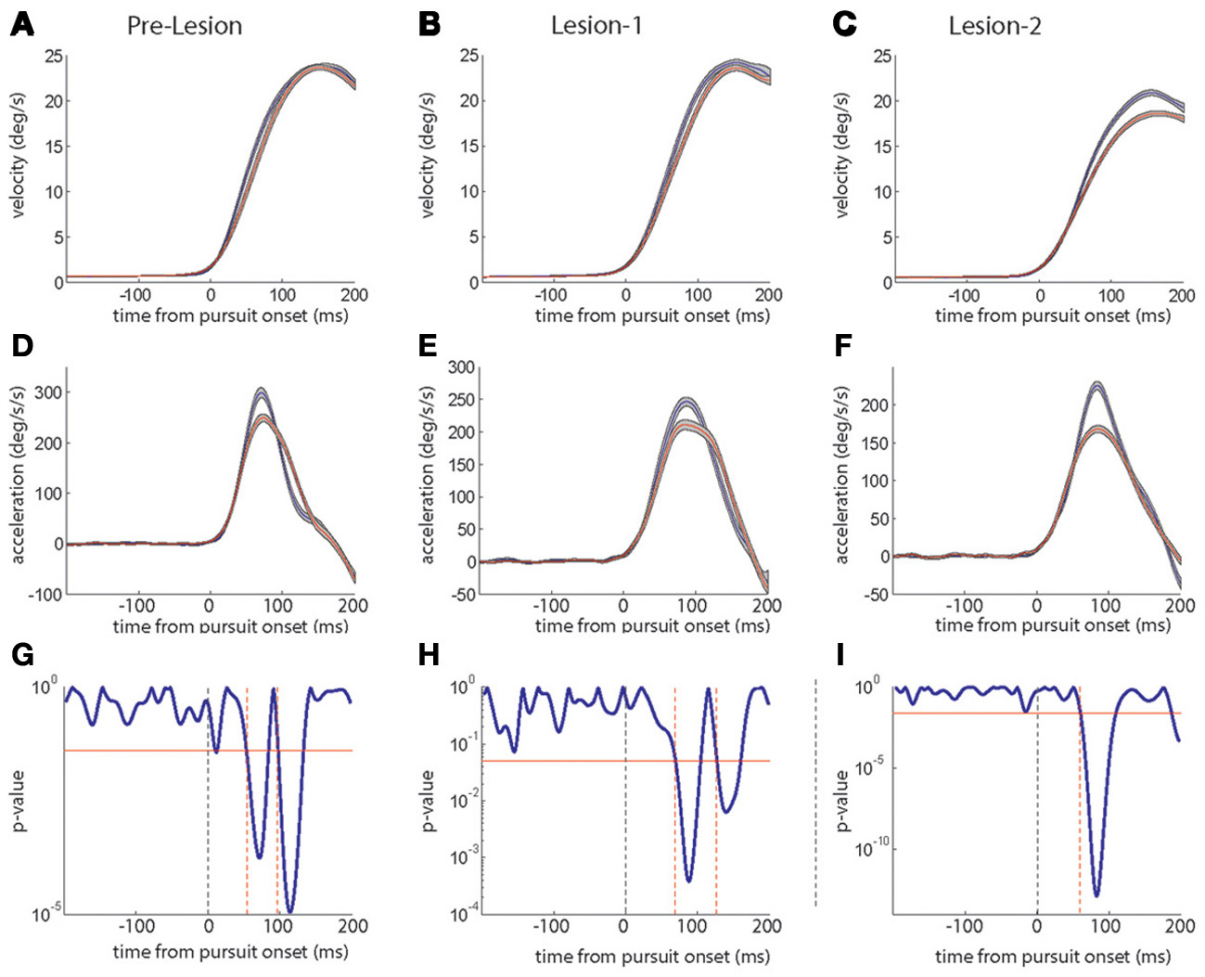
**Plots of average instantaneous eye velocity (top row) and eye acceleration (middle row) as function of time in a trial**. Blue traces give the averages for the first, red traces the averages for the last quartile of trials. The first column depicts the pre-lesion data, the middle column data collected 20 days after L1 and the right column shows data acquired 45 days after L2. The gray shadow around the red and blue traces signifies the standard error. The lower row panels **(G–I)** depict the periods when the acceleration profiles [blue and red; **(D–F)**] were significantly different from each other based on a running paired *t*-test. The red horizontal line indicates a *p*-value of 0.05. The dashed vertical (black) line marks pursuit onset and the red vertical lines show the time points when the significance level crossed the *p*-value of 0.05.

Figure 6 compares the average eye velocity traces for the first (blue) and the last quarter (red) of the trials. In accordance with the temporal averages discussed before, instantaneous eye velocity did not change in the course of experiments, neither pre-lesion (Figure 6A) nor after L1 (Figure 6B), as indicated by almost congruent velocity traces. However, after L2, the traces, indicating mean instantaneous eye velocity in the first and the last quarter exhibited a clear separation, due to the aforementioned drop in peak velocity in the course of the experiment (Figure 6C). Peak SPEM velocity in pre-lesion resilience experiments as well as after L1 remained constant despite a drop in peak acceleration by virtue of a compensatory increase in the duration of the acceleration (Figures 6D,E, respectively). These changes were reflected by significant differences in the comparison of the mean acceleration profiles for the first and the last quarter of trials by running paired *t*-tests. A significant difference between the early and late acceleration profiles appeared twice: an earlier difference indicating the drop in peak acceleration and a later difference indicating the expansion of the acceleration profile (Figures 6G,H). The difference between early and late acceleration profiles occurred slightly earlier in time in pre-lesion experiments as compared to experiments after L1 (Figures 6G,H; see dotted red lines).

Contrary to both pre-lesion and to post L1 experiments, there was no compensatory expansion of the acceleration profile counteracting a drop in peak acceleration after L2 (Figure 6F), although this drop was comparable in size to the drop observed pre-lesion and post L1. This uncompensated drop in peak acceleration following L2 fully accounted for the drop in peak velocity (Figures 5C, 6C). Unlike pre-lesion and after L1 (Figures 6G,H), after L2 a statistical difference between early and late acceleration profiles appeared only once indicating the drop in peak acceleration without a later statistically significant expansion of acceleration duration (Figure 6I).

**Figure 7:**
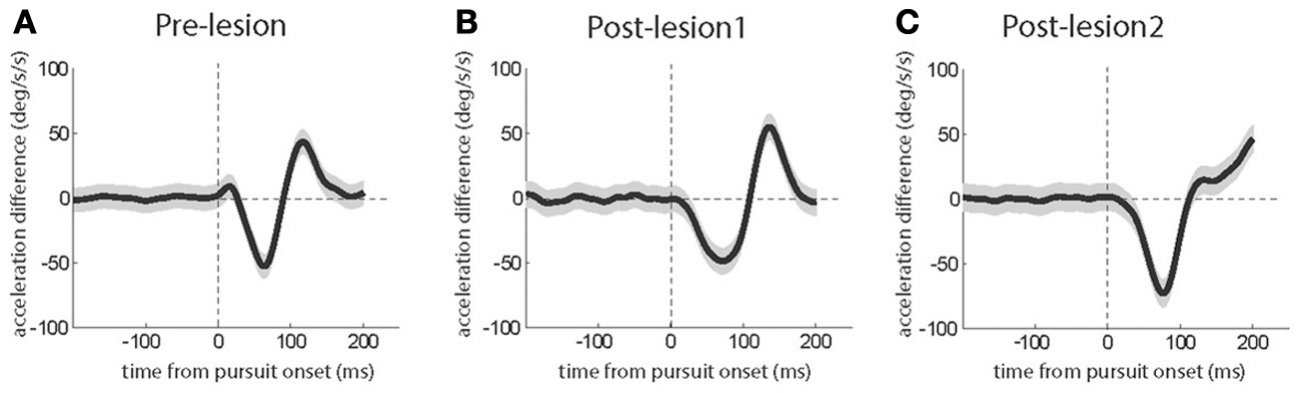
**Grand averages of eye acceleration profile differences based on a comparison of the first and the last quarter of trials in individual sessions before the lesions (A), after L1 (B) and after L2 (C)**. The gray shadows around the traces signify the standard error. The subject contributed 7 sessions before the lesion, 4 sessions after L1 and 6 sessions after L2.

To confirm the generality of the kinematic changes observed in the exemplary sessions discussed before, we calculated the differences between the mean acceleration profiles for the first quarter of trials and the last quarter of the trials for each session and averaged the resulting differences across sessions. Pursuit eye movement both in the pre-lesion period (Figure 7A) as well in the period following L1 (Figure 7B) exhibited an initial drop in acceleration difference which was followed by a later positive acceleration difference, the latter reflecting the expansion of the acceleration bursts. The positive acceleration difference was absent after L2 (Figure 7C).

## Discussion

Our behavioral observations on smooth-pursuit initiation suggest that gain-increase SPA is a consequence of maintaining a given level of acceleration for a longer time period and gain-decrease SPA results from a drop in peak acceleration, not accompanied by significant changes in the duration of the acceleration peak. Previous studies made similar observations for different velocity steps suggesting that kinematic changes associated with SPA prevail across a wide velocity range (Takagi et al., 2000; Dash et al., 2013). The novel aspect of this study is that we found that the changes in peak acceleration associated with the two forms of SPA mimic complementary features, characterizing usage dependent changes of smooth pursuit eye movement kinematics, observed if the pursuit system is challenged with the need to carry out a few hundred repetitions of stereotypic smooth pursuit eye movement trials (SPEM resilience experiment). Based on these similarities, we conclude that gain-decrease SPA is the manifestation of fatigue and gain-increase SPA reflects the functionality used to compensate fatigue.

Similar to gain-decrease SPA, a drop in peak acceleration is also exhibited by the eye movements in the later parts of the SPEM resilience experiment. We interpret this drop in peak acceleration as a consequence of *fatigue*, a term we use to capture short-term changes of the pursuit system due to usage. Pursuit fatigue seems to be analogous to saccadic fatigue, which is characterized by a drop in peak saccadic velocity. Saccadic fatigue is most probably a consequence of changes of the cognitive state of the observer, rather than a reflection of use-dependent changes of the oculomotor plant (Chen-Harris et al., 2008; Golla et al., 2008; Prsa et al., 2010), although an oculomotor component may play a role under specific conditions. Our study design and results cannot distinguish between the different potential causes of fatigue associated with pursuit initiation.

We suggest that the changes in eye movement acceleration observed during SPA and SPEM resilience may be attributed to the working of two independent processes: one—due to fatigue—modulates peak acceleration to decrease (gain-decrease SPA and SPEM resilience). The second one up-regulates acceleration duration, helping to compensate the loss of peak acceleration in the case of SPEM resilience, thereby ensuring a maintained initial eye velocity throughout the experiment. An up-regulation of the acceleration duration also underlies the increase in initial pursuit eye velocity in gain-increase SPA. We think that these two processes are independent as only the latter was affected by the oculomotor vermal lesion. Both together establish an acceleration-duration trade-off that ensures an adequate level of open-loop eye velocity. This acceleration-duration trade-off is comparable to the velocity-duration trade-off that has been established to govern saccades and their modification by short-term saccadic adaptation (STSA) (Catz et al., 2008; Golla et al., 2008; Xu-Wilson et al., 2009; Prsa et al., 2010). During gain-decrease STSA, the saccade amplitude gets smaller because peak saccade velocity declines without being accompanied by relevant changes in saccade duration. On the other hand, gain-increase STSA is a consequence of an increase in saccade duration, not accompanied by major changes in peak velocity and, finally, saccadic fatigue—the loss in peak velocity due to usage—is compensated by an up-regulation of saccade duration. The intriguing formal correspondence of the kinematic adjustments associated with SPA and STSA, respectively, as well as the consequences of fatigue for both types of visually guided eye movements suggests that both may rely on the same neuronal machinery, warranting the precise velocity-duration/acceleration-duration trade-off needed. As indicated by the consequences of lesioning lobuli VI and VII, this trade-off depends on the integrity of this specific part of the cerebellum. Furthermore, after cerebellar disease involving the vermis this ability to maintain saccade accuracy is lost due to a loss of the ability to increase saccade duration by expansion of the velocity profile (Golla et al., 2008; Xu-Wilson et al., 2009). Actually, lesions of the cerebellar OMV destroy STSA (Takagi et al., 1998; Barash et al., 1999; Golla et al., 2008) as well as SPA (Takagi et al., 2000). This is concordant with the fact that the SPA as well as STSA lead to specific changes in OMV purkinje cells (PCs). OMV houses both PCs affected by SPA as well as PCs responding to STSA (Catz et al., 2008; Kojima et al., 2010; Dash et al., 2013).

In summary, our study suggests that the OMV deploys a common mechanism—the precise adjustment of movement time (duration of velocity profile during saccades and duration of acceleration profile during SPEM)—in order to compensate fatigue in both types of goal-directed eye movements, saccades and SPEM.

### Conflict of interest statement

The authors declare that the research was conducted in the absence of any commercial or financial relationships that could be construed as a potential conflict of interest.
